# A review of common immunotherapy and nano immunotherapy for acute myeloid leukemia

**DOI:** 10.3389/fimmu.2025.1505247

**Published:** 2025-03-10

**Authors:** Yaoyao Wang, Xiancong Yang, Yalin Liu, Youjie Li

**Affiliations:** ^1^ Department of Pediatrics of Yantai Affiliated Hospital, The Second Clinical Medical College of Binzhou Medical University, Yantai, Shandong, China; ^2^ Department of Biochemistry and Molecular Biology, Binzhou Medical University, Yantai, Shandong, China; ^3^ Laboratory Department, Qilu Hospital of ShanDong University Dezhou Hospital, Dezhou, Shandong, China

**Keywords:** acute myeloid leukemia, antibody therapy, CAR-T cell therapy, immune checkpoint inhibitors, nanoimmunotherapy

## Abstract

Acute myeloid leukemia (AML) is a highly aggressive hematological malignancy. Traditional chemotherapy methods not only bring serious side effects, but also lead to high recurrence rate and drug resistance in some patients. However, as an emerging therapeutic strategy, immunotherapy has shown great potential in the field of AML treatment in recent years. At present, common immunotherapy methods for AML include monoclonal antibodies, CAR-T cell therapy, and immune checkpoint inhibitors. With the deepening of research and technological progress, especially the application of nanotechnology in medicine, new immunotherapy is expected to become one of the important means for the treatment of acute myeloid leukemia in the future.

## 1 Introduction

Acute myeloid leukemia (AML) is a hematological malignancy that is prevalent worldwide. Its main feature is that myeloid progenitor cells or primitive granulocytes cannot differentiate normally, resulting in abnormal proliferation, accompanied by fever, anemia, bleeding and bone pain and other clinical (1, 2). With the in-depth study of the pathogenesis of AML, the prognosis of patients with acute myeloid leukemia has improved, but AML is still one of the cancer types with high recurrence rate and mortality (3–5).

The treatment of acute myeloid leukemia mainly uses chemotherapy, radiotherapy and hematopoietic stem cell transplantation, among which chemotherapy is regarded as the primary treatment. However, the chemotherapy methods have remained basically unchanged for decades, and the efficacy and prognosis are not optimistic. Most patients have failed to achieve complete remission or disease recurrence (6, 7). Immunotherapy has become an important research direction in the treatment of acute myeloid leukemia. The immune system plays an important role in cancer treatment, and immunotherapy has been widely used in B cell carcinoma and various solid cancers (8, 9). Common immunotherapy includes monoclonal antibodies, antibody-drug conjugates, radionuclide conjugates, bispecific antibodies, and chimeric antigen receptor T cells (CAR-T cells) (10, 11).

The research of new immunotherapy using nanotechnology in the field of cancer treatment has become a hot topic, and exploring the application of nanotechnology in cancer treatment has become a quite popular task (12). The rapid development of nanotechnology has promoted the research and application of nanomaterials and nanoparticles (NP). These tiny substances have shown broad application prospects in the fields of biology and medicine. As an ideal carrier for targeted drug delivery systems, nanoparticles can overcome the problem of tumor drug resistance caused by biochemical and physical barriers and cellular and non-cellular mechanisms in traditional chemotherapy (13).

**Figure 1:**
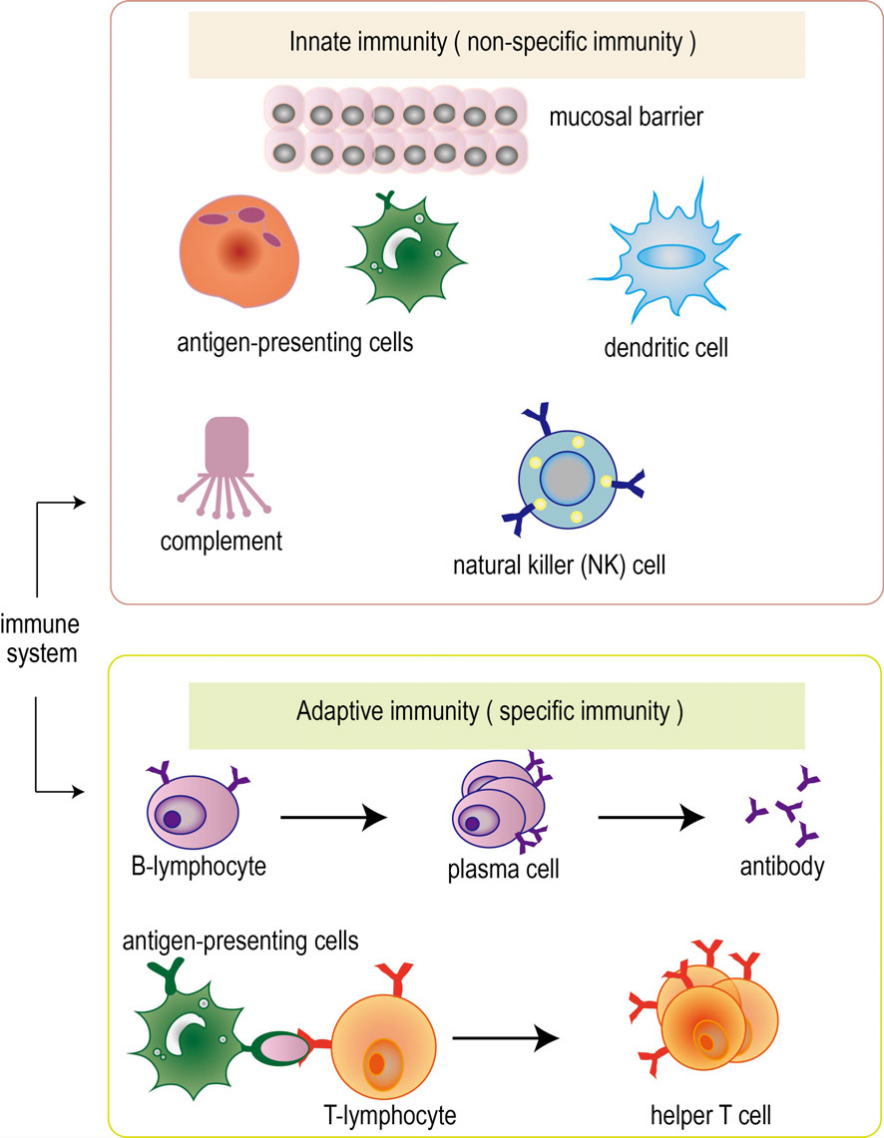
Classification of immune system. Immune system can be divided into innate immunity (also known as non-specific immunity) and adaptive immunity (also known as specific immunity). In adaptive immunity, it can be further subdivided into humoral immunity and cellular immunity. The immune system performs its function through the synergy of multiple immune cells.

The immune system can be divided into innate immunity (also known as non-specific immunity) and adaptive immunity (also known as specific immunity) (**Figure 1**). In adaptive immunity, it can be further subdivided into humoral immunity and cellular immunity. Innate immunity is the earliest immune response produced by human beings, which protects the human body through skin, mucosa, macrophages and natural killer cells. Adaptive immunity is an immune response produced after the first contact with pathogens, which can identify and eliminate pathogens that infect the human body (14, 15). The process of adaptive immunity includes four stages: perception, recognition, activation and execution. First, the body perceives the invading pathogens and recognizes and resists pathogens through immune cells and systems on the body surface and mucosa. The process of recognition is to produce specific antibodies and T cells to identify and attack foreign pathogens through the interaction of humoral immunity and cellular immunity. The process of activation is that immune cells release cytokines to regulate and activate other immune cells to form an immune response. The process is carried out by T cells and B cells synergistically to attack and eliminate pathogens by producing antibodies and cell-mediated effects (16, 17).

There are a variety of immune cells and immune molecules in the human immune system that can resist the occurrence of cancer. For example, natural killer (NK) cells can directly recognize and kill cancer cells (18); T cells recognize and attack cancer cells by recognizing specific proteins on the surface of cancer cells; antibodies produced by B cells can specifically target and neutralize cancer cells. In addition, cytokines such as interferons and interleukins can promote the activation of immune cells and enhance their ability to target cancer cells. Chemokines can attract immune cells to the tumor site, and antibodies can bind to cancer cells and label them to be destroyed by immune cells. However, through development, cancer has been able to avoid being recognized by the immune system, so cancer immunotherapy can stimulate the patient ‘s immune system to recognize or destroy abnormally proliferating cells (19). In addition, with the emergence of nanotechnology, we are expected to inhibit tumor growth, reduce chemotherapy resistance and prevent metastasis, which provides new ideas and strategies for tumor immunotherapy.

## 2 Immune escape mechanism of acute myeloid leukemia

**Figure 2:**
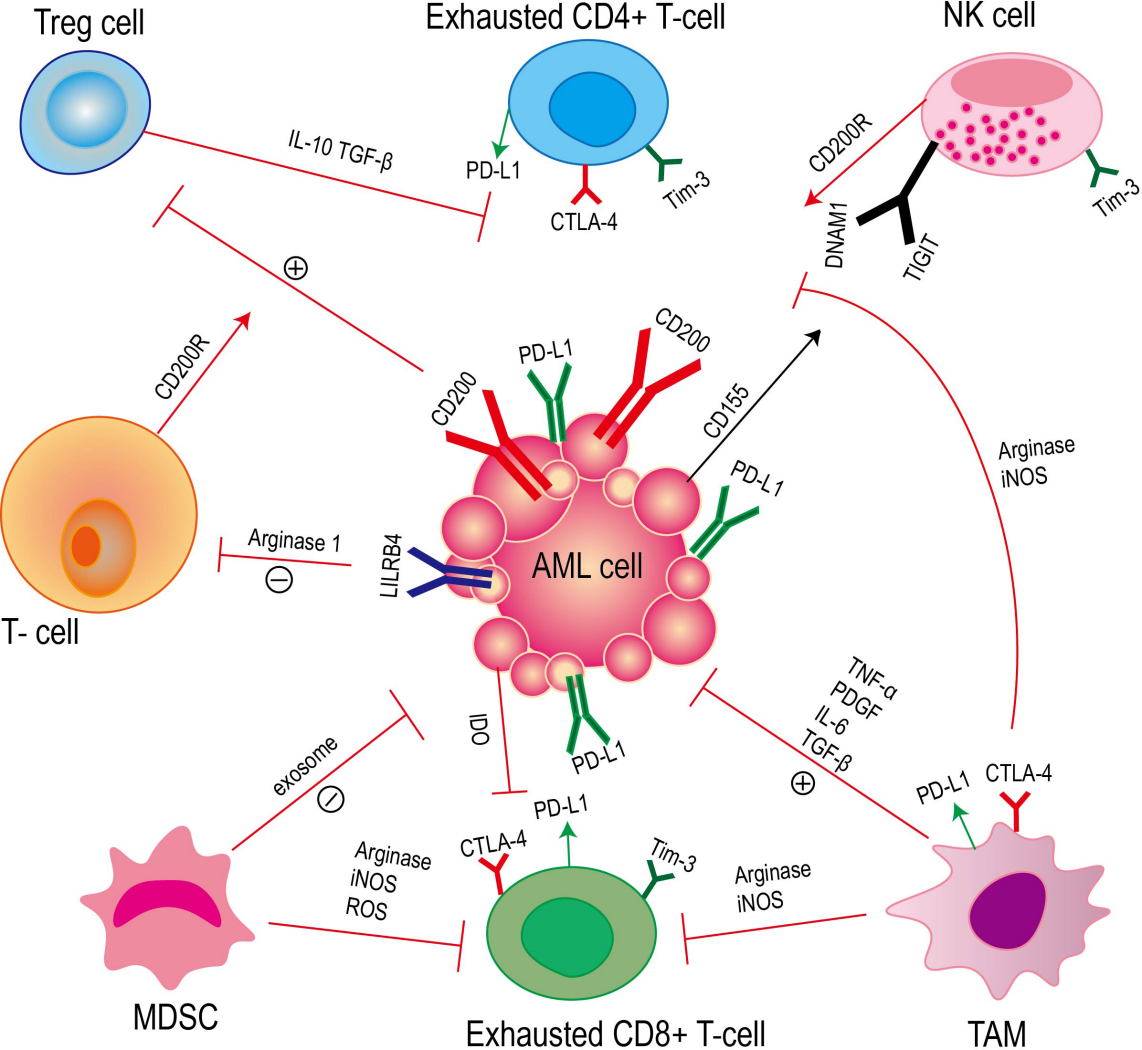
Acute myeloid leukemia achieves immune escape through the interaction between immune cells and immune factors. This process mainly involves T cells, natural killer cells (NK cells), regulatory T cells (Tregs), myeloid-derived suppressor cells (MDSCs) and tumor-associated macrophages (TAMs), as well as cytokines and related signaling pathways secreted by these cells.

Acute myeloid leukemia cells evade the recognition and clearance of the immune system by constructing an immunosuppressive tumor microenvironment. The formation of this tumor microenvironment involves a variety of immune cells and immune factors (20, 21) (**Figure 2**).

T cells play a crucial role in the immune response. The interaction between the expression of AML surface receptors (leukocyte immunoglobulin-like receptor B4, LILRB4) and T cells leads to the inhibition of T cell proliferation. However, LILRB4 directly inhibits T cell proliferation and cytotoxicity by mediating the release of arginase-1 (22). CD200 is a type I membrane glycoprotein of the immunoglobulin superfamily, which is up-regulated on the surface of AML cells (23). The interaction of CD200 with T cell CD200 receptor (CD200R) leads to an increase in regulatory T cells (Tregs) and a decrease in memory T cell function (24). Studies have confirmed that compared with healthy individuals, the number of CD4 + and CD8 + T cells in AML patients is significantly reduced, and these cells also show the characteristics of aging (25). At the same time, the high proportion of lymphocytes and T lymphocytes in bone marrow is related to the improvement of survival rate of AML patients (26).

NK cells are a kind of congenital lymphocytes (27). NK cells can effectively identify and eliminate AML cells *in vivo*, which can prevent the occurrence of diseases. NK cells regulate the immune response by secreting tumor necrosis factor-α (TNF-α) and interferon-γ (IFN-γ). Interferon-γ can promote the maturation of dendritic cells, thereby promoting the formation of adaptive immunity (28). In AML blasts including leukemia stem cells (LSC), the up-regulation of the surface glycoprotein CD200 of immunosuppressive cells is associated with the functional inhibition of natural killer (NK) cells through their receptor CD200R (29, 30). In the DNAM1/TIGIT/CD96 signaling pathway, DNAM1 and TIGIT act as receptors for activating and inhibiting NK cells, respectively, and both target CD155, which is expressed in AML blasts. The relative expression levels of ligands and receptors in this signaling pathway are different, and this change in expression is related to the inhibition of NK cell function (31, 32).

Regulatory T cells (Tregs) play an immunosuppressive role in AML. In AML, Tregs are recognized as a factor that can be used by leukemia cells to evade immune monitoring (33). Studies have shown that in AML, the interaction between PD-1 and PD-L1 can promote the inhibition of Teffs by Tregs, thereby weakening the anti-tumor immune response (34). At the same time, Tregs can secrete immunosuppressive factors (such as TGF-β and interleukin-10) or direct cell contact to inhibit the effector T cell response and evade the recognition of the immune system (35–37). The number of Tregs with high inhibitory activity was increased in AML patients at diagnosis, suggesting that these cells may play an important role in the anti-tumor immune response (38). Recent studies have shown that Tregs are more abundant in the bone marrow of AML patients and are beneficial to the growth of leukemia cells. The large number of Tregs is not conducive to the treatment of AML patients (39). At the same time, a large number of studies have shown that the depletion of Treg cells in the tumor microenvironment can enhance the host ‘s anti-tumor immunity (40–42).

Myeloid-derived suppressor cells (MDSC) inhibit immune cell response through various mechanisms in AML. Myeloid-derived suppressor cells (MDSC) induce T cell tolerance in AML patients through a variety of mechanisms, such as PD-L1, arginase, indoleamine 2,3-dioxygenase (IDO), TGF-β and IL-10 (43–46). MDSC can directly inhibit anti-tumor T cell response, and MDSC can also indirectly inhibit antigen-specific T cell activation by inhibiting the function of antigen-presenting cells (APC). The presence of MDSC can effectively block Ag-specific CD8-mediated T cell function (47, 48). MDSC mainly exerts T cell inhibition by expressing ARG1, iNOS and ROS (49–51). Studies have shown that MDSC stops the cell cycle of T cells and blocks T cell proliferation, rather than directly killing T cells (52). MDSC also secretes various exosomes that promote tumor growth, which are transported to the tumor site and induce immunosuppression (53). In general, the mechanisms of MDSC in promoting tumor immunosuppression mainly include:1) reducing the amino acids required for T cell proliferation and activation; 2) To release immunosuppressive cytokines and promote the differentiation of regulatory B (Breg) cells and regulatory T cells (Treg); 3) Recruiting regulatory T cells; 4) binding to the inhibitory receptor PD1 to block the killing function of T cells/NK cells; 5) down-regulation of NK cell activating receptors; 6) Down-regulated STAT-3 and increased HIF1α to induce M2 macrophage differentiation; 7) Secretion of S100A8/9 promotes the polarization and chemotaxis of MDSC and M2 macrophages in the tumor microenvironment; 8) Inhibition of antigen presenting function of dendritic cells (DC) (54).

Tumor-associated macrophages (TAMs) play a key role in the immunosuppressive microenvironment of acute myeloid leukemia (AML). TAMs are derived from bone marrow mononuclear cells, including M1 macrophages with anti-tumor function and M2 macrophages with tumor-promoting properties (55). Studies have shown that there is a significant correlation between TAM and poor prognosis and recurrence of cancer patients (56). TAM can promote tumor formation and help tumor cells avoid being attacked by the body ‘s immune system by secreting growth factors and cytokines that support tumor cell proliferation, such as tumor necrosis factor (TNF)-α, platelet-derived growth factor (PDGF), TGF-β and IL-6 (57–63). TAMs can also regulate the killing function of T cells and NK cells (64).

For example, M1 macrophages regulate the immune microenvironment to activate NK cells, leading to apoptosis and tissue fibrosis (65). In malignant pleural mesothelioma, TAMs mainly have M2 phenotype, and there is a negative correlation between TAMs and T cells (66). In AML cells, ICOSL expression leads to the expansion of ICOS + Tregs, thereby promoting immune escape, and IL-10 secreted by ICOS + Tregs promotes the proliferation of AML cells (67). In addition, studies have shown that tumor-associated macrophages (TAMs) can directly inhibit the function of T cells by expressing immune checkpoint molecules such as cytotoxic T lymphocyte antigen 4 (CTLA-4) and programmed death 1 (PD-1) (68–70). Most patients with acute myeloid leukemia (AML) lack arginine succinate synthase-1 (ASS1), which leads to a decrease in arginine synthesis. However, the depletion of extracellular arginine promotes macrophage polarization to M2 (71).

## 3 Common immunotherapy for acute myeloid leukemia

**Figure 3:**
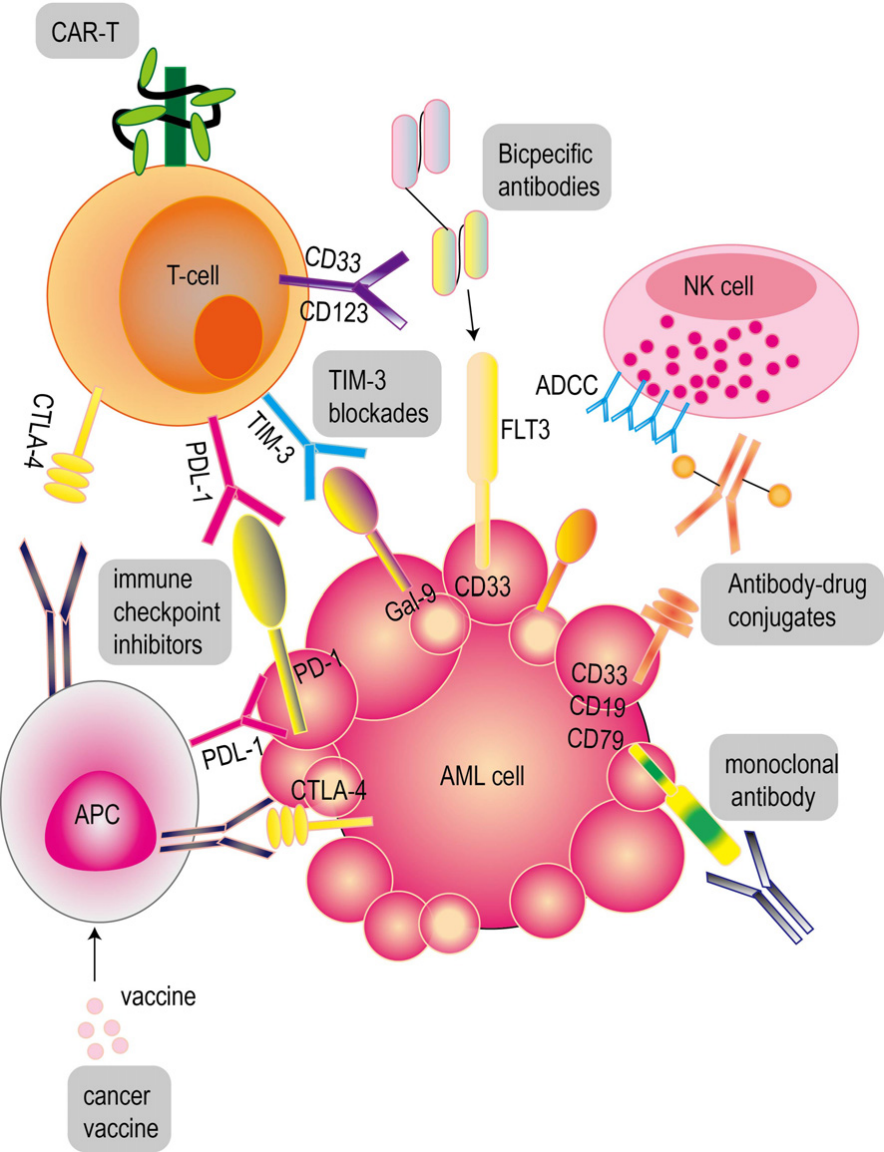
Common immunotherapy for acute myeloid leukemia. These therapies include antibody immunotherapy (monoclonal antibodies, bispecific antibodies, antibody-drug conjugates), CAR-T cell therapy and cancer vaccines for acute myeloid leukemia. PD-1 and CTLA-4 inhibitors are common immune checkpoint inhibitors (ICIs).

Immunotherapy shows potential ability to overcome relapse and drug resistance, which is particularly critical for patients with relapsed or refractory AML. These therapies play a key role in the treatment of AML and show broad prospects (**Figure 3**).

### 3.1 Antibody immunotherapy for acute myeloid leukemia

Antibody is a kind of protective protein secreted by plasma cells under the stimulation of antigen. The function of antibodies in acute myeloid leukemia depends on the following aspects: First, after the antibody binds to the surface antigen of tumor cells, it uses its Fc domain to recruit immune effector molecules, thereby triggering natural killer (NK) cell-mediated antibody-dependent cytotoxicity (ADCC), antibody-dependent phagocytosis or complement-dependent cytotoxicity; secondly, through receptor-mediated endocytosis of antibody-drug conjugates (ADCs) and radionuclide conjugates, it can effectively deliver toxic carriers to kill leukemia cells. The third is to use bispecific or multispecific antibodies to bind these cells to target leukemia cells, thereby enhancing the anti-leukemia effect of T cells or NK cells.

Since most leukemia cells are associated with the positive expression of specific antigens (such as CD123, CD33, CD96 and CLL-1) (72), targeted immunotherapy, including monoclonal antibodies (73) and bispecific antibodies, is currently in the clinical evaluation stage. These therapies aim to target multiple antigens expressed on AML blast cells, of which CD33 and CD123 are the most common targeted antigens (74). The bispecific T cell conjugative molecule (BiTE) is composed of a single variable fragment (Fv). Bispecific T cell conjugator (BiTE) targeting CD33 can activate and amplify T cells in autologous clinical samples of patients with AML, and mediate the lysis of primary AML cells and normal myeloid cells *in vitro* in a dose-dependent manner (75–77). The use of anti-CD19 BiTE triggered CD33-independent activation, resulting in CD33 expression on a small number of T cells. This phenomenon is related to the self-killing of T cells, but has little effect on the function of cytotoxic T cells associated with AML (78). The single-chain Fv trisomics (sctb) based on CD16 single-chain Fv fragment (scFv) targeting CD123 exhibits significant anti-leukemia activity, but its effect is not as good as the dual-targeting sctb targeting both CD33 and CD123 (79). 7370 anti-FLT3 bispecific IgG can activate T cells in patients with acute myeloid leukemia, thereby inducing cytotoxicity to autologous blasts. The results of *in vivo* experiments showed that the bispecific antibody could also effectively guide human T cells to target AML cell lines and produce killing effects *in vivo*. This effect depends on the expression of FLT3 antigen on the surface of AML cells, and is not affected by FLT3 mutation status. Both double-targeted single-chain Fv trisomy (sctb) and single-targeted sctb use isolated monocytes (MNC) as effector cells, which can effectively induce antibody-dependent cytotoxicity (ADCC) of two different acute myeloid leukemia (AML) -derived CD33 and CD123 double-positive cell lines at low concentrations (80). In addition, a bispecific single-chain Fv (bsscFv) was formed by binding another scFv that is specific for low-affinity Fcγ receptor III (CD16). This bsscFv can effectively mediate the cleavage of AML-derived leukemia cell lines in a specific concentration of ADCC reaction (81). Bispecific antibodies produced by chemical coupling of anti-CD3 and anti-CD13 Fab ‘ fragments enhance the cytotoxicity of peripheral blood mononuclear cells (PBMC) to CD13-positive AML cells stimulated by IL2 or IL7 (82). At the same time, the clinical potential of monoclonal antibodies (mAbs) against CD123 in the treatment of AML has been supported by relevant evidence (83). BI836858 is an antibody against CD33 with an Fc domain. Decitabine can increase the expression of NKG2D ligands on the surface of leukemia cells, thereby enhancing BI836858-mediated antibody-dependent cell-mediated cytotoxicity (ADCC) (84). Evorpacept (ALX148) is mainly composed of a modified SIRPαD1 domain targeting CD47 and can bind to the inactivated human IgG1 fragment (Fc) (85). At present, the molecule is being studied in an ongoing phase I/II clinical trial (NCT04755244) to evaluate its efficacy in combination with the BCL2 inhibitor venetox and AZA, especially for patients with relapsed/refractory acute myeloid leukemia (R/R AML) who have not been treated or are not suitable for standard induction chemotherapy (86). At the same time, *in vivo* experimental results showed that under the action of activated human T cells, AMG 330 targeting CD33 could inhibit the growth of xenografts of humanized mouse subcutaneous AML cell lines, thereby significantly improving the survival rate (76, 77). In addition, treatment with anti-CD28 activation antibody not only enhanced the killing effect of AMG 330 on human AML cell lines, but also increased the cytotoxicity of primary AML samples from patients with refractory leukemia (87). Lintuzumab (SGN-33) is a monoclonal antibody (mAb) against CD33. Studies have shown that lintuzumab may have an anti-leukemia effect, which can induce remission after cytoreductive surgery with low-dose cytarabine in untreated AML patients (88). Studies on AML animal models have shown that MGD024 combined with cytarabine or venetox can almost completely eliminate tumor cells. Therefore, a drug dose escalation study was proposed to evaluate the safety of MGD024 in refractory/recurrent hematological malignancies (including AML and BPDCN).

A few years ago, researchers developed an antibody-drug conjugate (ADC) treatment strategy, which showed good therapeutic effect and ideal side effects (89). ADC has gradually become an emerging chemotherapeutic drug for the treatment of cancers including AML (90). IMGN632 is an antibody-drug conjugate (ADC) targeting CD123, which uses a new IGN payload (91). In addition, compared with X-ADC, IMGN632 showed cytotoxic effects on AML samples at doses that had no adverse effects on normal myeloid progenitor cells (92). Clinical studies have shown that IMGN632 and venetoclax (a BCL-2 inhibitor for patients with acute myeloid leukemia) combined with azacitidine showed a consistent anti-leukemia synergistic effect, and the synergistic anti-leukemia effect between IMGN632 and venetoclax was verified in leukemia cell lines and xenotransplantation (PDX) derived from patients with acute myeloid leukemia. Mylotarg is a kind of ADC, which is used to treat newly diagnosed or refractory CD33 positive AML. Compared with Mylotarg, ADC targeting AML-specific antigens is expected to provide a more effective treatment for AML, and has a wider range of indications (93). SGN-CD123A is an antibody-drug conjugate (ADC) based on humanized CD123 antibody, which can effectively induce the apoptosis of leukemia cells expressing CD123. The drug shows a significant effect on promoting the apoptosis of leukemia cells in myeloid leukemia cell lines, primary AML blasts and patient-derived xenograft models (94). Antibody therapy has made significant progress in the clinical research of acute myeloid leukemia, but it still needs to be continuously optimized and improved to provide more effective and safe treatment for patients.

### 3.2 Immune checkpoint inhibitors

Immune checkpoint inhibitors (ICIs) relieve the inhibition of tumor cells on the immune system by interfering with the signal transmission mechanism between tumor cells and immune cells, thereby enhancing the immune system ‘s ability to attack tumor cells. Specifically, immune checkpoint inhibitors can effectively kill tumor cells by blocking the inhibitory molecules on the surface of T cells and transforming T cells from an ‘ exhausted ‘ state to an ‘ activated ‘ state, which has been proved to be a promising therapeutic option (95). But ICIs are not very promising drugs in AML treatment, and can be consider only as an addition to the treatment, do not work in monotherapy. In the current study, PD-1 and CTLA-4 are the two most active checkpoint receptors in the current study. They play a key role in different stages of anti-tumor immune response. At the same time, inhibition of CTLA-4 and PD-1 is the most widely used two immune checkpoint blocking strategies in clinical practice.

PD-1 inhibitors commonly used in clinical research for the treatment of AML include drugs such as ipidilizumab, nivolumab, pembrolizumab, durvalumab and atezolizumab (96). The results showed that in patients with AML, the complete remission rate (CR) of anti-PD-1 antibody nivolumab combined with azacitidine was 18%, and the hematological improvement rate was 15% (97). Studies have shown that the combination of azacitidine, nivolumab and ipilimumab (an anti-CTLA-4 antibody) has achieved complete remission (CR) or complete remission with incomplete hematological recovery (CRi) in 3% of patients with AML (98). The application of nivolumab combined with conventional induction chemotherapy (such as idarubicin plus cytarabine) in newly diagnosed AML patients is feasible (99). The efficacy of Pembrolizumab combined with decitabine or azacitidine in patients with R/R AML was similar to that of azacitidine combined with nivolumab. Tiragolumab is an anti-TIGIT antibody that can improve the prognosis of lung cancer patients when combined with atezolizumab. Anti-TIGIT antibody reshapes the tumor microenvironment by enhancing the blocking effect of PD-L1 on bone marrow cells and Treg cells, thereby improving the prognosis of tumor patients. The study found that tumor patients with higher baseline levels of macrophages and regulatory T cells in tumors had a better prognosis when treated with atezolizumab combined with tiragolumab, while atezolizumab alone did not have this effect. This indicates that TIGIT checkpoint inhibitors can play a role by reshaping the immunosuppressive tumor microenvironment (100). And the study reported the safety of azacitidine combined with duralizumab in patients with MDS and AML.

CTLA-4 (CD152) transmits immunosuppressive signals to terminate the immune response by interacting with CD80 and CD86 ligands. In the study of the treatment of melanoma patients, we found that anti-CTLA-4 antibody ipilimumab can effectively increase the proportion of Teff/Tregs, enhance the activity of NK cells, and restore the function of T effector cells, thereby significantly prolonging the survival of patients (101). Anti-CTLA-4 can enhance the function of AML-specific T cells, which is manifested in increasing its frequency, cytotoxicity and IFN-γ secretion (102). When ipilimumab monotherapy was used in patients undergoing hematopoietic stem cell transplantation (HCT) for recurrent hematological malignancies, a complete remission (CR) rate of 23% and a partial remission (PR) rate of 9% were observed (103). A phase I clinical trial for recurrent or refractory AML and myelodysplastic syndrome (MDS) is underway to evaluate the efficacy of ipilimumab in combination with decitabine (DAC) in patients who have received or have not received allogeneic hematopoietic stem cell transplantation (HCT). At present, the trial has not yet begun to recruit subjects (clinical trial registration number: NCT2890329).

### 3.3 Chimeric antigen receptor T cell therapy

The concept of chimeric antigen receptor (CAR) T cell therapy was first proposed in 1993 (104). This therapy has achieved remarkable results in the treatment of lymphatic and hematological malignancies by targeting unique targets such as CD19, CD22 and BCMA (105). CAR-T cells are genetically engineered from autologous peripheral blood T cells and allogeneic CAR-T cells. These cells have specific extracellular antigen recognition domains, which are usually composed of single-chain variable fragments of monoclonal antibodies and are connected to the intracellular signal transduction domain (106). The advantages of chimeric antigen receptor (CAR) T cell therapy are reflected in the following aspects: (a) CAR-T cells are activated only when recognizing specific targets (107); (b) CAR-modified cells can be effectively eliminated after disease eradication by using non-persistent cell types or safe switching mechanisms. The idea of safety switch is to trigger it when a high grade of CRS occurs to save the patient’s life; if the patient is safe without a serious toxicity, keeping CAR T cells in the body provides the treatment for many more years after disease eradication; the CAR T cells not active sitting in the body, ready to attack the cancer cells again when needed. “The safety switch” works like “an insurance policy” for the patient, in case of the relapse of the disease, high T cells activation followed by a high grade of CRS. (c) Genome editing of hematopoietic stem cells (HSCs) to generate hematopoietic systems that are resistant to CD33 (destruction of normal bone marrow) (108).

**Figure 4:**
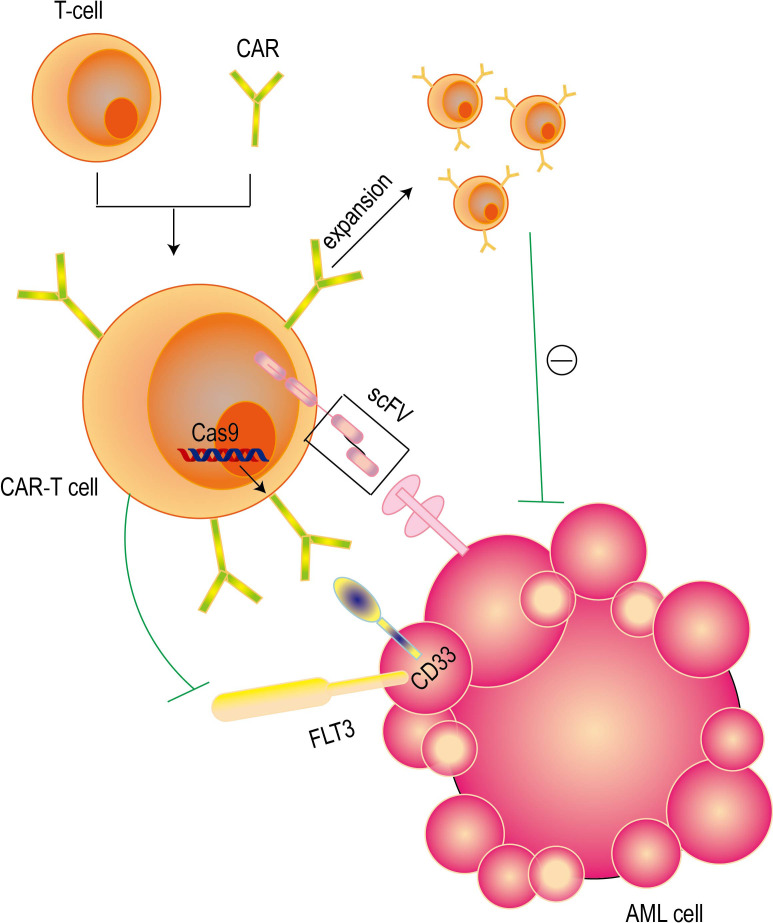
Mechanisms of CAR-T cell therapy. CAR-T cell therapy achieves precise targeted elimination of AML cells by combining the killing ability of T cells and the high specificity of antibodies. Single-chain variable fragments (ScFv) give this therapy the ability to accurately identify targets, while the intracellular signaling domain is responsible for activating the cytotoxic effects of T cells. At the same time, CRISPR-Cas9 technology can be used to perform gene ablation on CD33 antigen to kill AML cells.

At present, a variety of strategies have been developed to improve the safety and reliability of CAR-T cell therapy in AML treatment (**Figure 4**) (**Table 1**). These strategies include using inducible caspase-9 to control the death of CAR-T cells and adjusting the affinity of CAR-T cells to only target cells with high expression levels (109). A large number of studies have shown that anti-CD123 CAR-T cells exhibit significant anti-leukemia activity before clinical practice. However, some studies have also raised concerns about the toxicity of hematopoietic stem cells (HSCs) (110). A variety of anti-CD33 CAR-T cell constructs have shown significant preclinical therapeutic effects on primary acute myeloid leukemia (AML) cells *in vitro* and in humanized animal models, and their toxic effects on leukemia cells and non-leukemia myeloid cells have also been observed (111). At present, in a clinical trial report of anti-CD33 CAR-T cell therapy, the treatment of a patient with refractory acute myeloid leukemia (AML) was recorded in detail. After receiving autologous anti-CD33 CAR-T cell infusion, the patient not only developed cytokine release syndrome (CRS), but also observed a temporary decrease in the original cells in the bone marrow (BM) (112). Studies have shown that human peripheral blood T lymphocytes transduced with CD19 CAR can completely eliminate lymphoma and leukemia in immunodeficient mice (113). CAR-T cells targeting CD19 in combination with T cell inhibitors can destroy pathological B cells and regulate T cell response, thereby inducing remission of refractory antisynthase syndrome (114). In a phase I clinical trial (ClinicalTrials.gov identifier:NCT02842138) for patients with B-cell lymphoma,11 patients received CD19-BBz (86) CAR-T cell therapy. Each patient received a dose of 2×10^8^ to 4×10^8^ cells of CD19-BBz (86) CAR-T cells, of which 6 patients achieved complete remission (115). A study showed that 23 of 27 adult and pediatric ALL patients (including 11 patients with extramedullary diseases) achieved complete remission after the first CD19 CAR-T treatment (116). CAR-T cell therapy has been reported to show great potential in improving the treatment of relapsed or refractory B-cell malignancies (117–119). According to relevant reports, a bispecific split CAR (BissCAR) T cell targeting CD13 and TIM-3 has a significant effect in clearing patient-derived acute myeloid leukemia (AML). At the same time, in mouse and patient-derived xenograft models, this treatment method is less toxic to normal hematopoietic stem cells (HSC), myeloid cells, and healthy organ systems (120). CD123 CAR-T cells have shown significant anti-leukemia effects *in vitro* on leukemia cell lines and primary patient leukemia cells, as well as *in vivo* using leukemia mouse models. However, it is unclear whether CD123 CAR-T cells affect the normal hematopoietic function of bone marrow (121, 122). A study selected ADGRE2 and CLEC12A as the target antigens by analyzing samples from patients with resistant AML and doing a series of tests in mouse models beginning in 2018 (123). They showed that CAR T cells designed for these antigens were safe and effective against human AML cells implanted in mice. Based on these results, the clinical trial was approved to go forward.CRISPR-Cas9 technology has been proved to be feasible for gene ablation of CD33 antigen in human HSPCs, and this technology shows multi-lineage hematopoietic recovery ability in *in vivo* model system (124). Based on this result, we will initiate a clinical trial that will combine allogeneic HSCT using transgenic CD33-negative HSCs and CD33-targeted CAR-T cell therapy (125). In addition to CD33 and CD123, targets such as folate receptor β (126), FLT3 (127), NKG2D ligand (128) and CD70 (129) are also being tested for the development of CAR-T cells.

**Table 1:** Clinical trial of CAR-T in the treatment of acute myeloid leukemia.

Study identifier	Phase	Start dates	Status	Target	Study population	Number (n)	Study Type
NCT04766840	I	2021-03-01	Unknown		R/R AML	9	Interventional
NCT03612739	I	2018-12	Withdrawn		AML/MDS		Interventional
NCT05672147	I	2023-12-07	Recruiting	CD33	R/R AML	27	Interventional
NCT05984199	I/II	2023-12-11	Recruiting	CD33	AML	24	Interventional
NCT04169022	Not Applicable	2019-07-10	Completed	IL1RAP	R/R AML	86	Interventional
NCT02159495	I	2015-12-15	Active, not recruiting	CD123	R/R AML	31	Interventional
NCT04692948	Not Applicable	2019-12-09	Unknown	CD276	R/R AML	5	Interventional
NCT06492304	I/II	2024-08-13	Recruiting	CD70	R/R TCL,BCL,AML	290	Interventional
NCT05017883	Not Applicable	2021-07-01	Recruiting		R/R FLT3 positive AML	5	Interventional

The latest research provides strong evidence for the effectiveness of the combination therapy of anti-FLT3 CAR-T cells and crenolanib. It has been found that pretreatment with crenolanib can significantly increase the expression level of FLT3 on the cell surface, thereby enhancing the ability of T cells to recognize AML cell lines. This effect has been observed in both *in vitro* experiments and animal models (130). The efficacy and safety of CAR-T therapy in AML are not convincing enough, which is mainly due to the lack of ideal therapeutic targets, a series of complex factors related to AML microenvironment, and CAR-T cell exhaustion (131). At present, the most promising candidate targets for CAR-T treatment of AML include CD33, CD123 and CLL1.A large number of literatures have reviewed these targets and other possible targets in detail (132). The resistance of AML to CAR-T therapy involves a variety of mechanisms, including antigen escape, inhibition of tumor immune microenvironment, CAR-T cell dysfunction, and tumor heterogeneity. In response to these challenges, CAR-T cells that simultaneously target multiple antigens can be developed to reduce the risk of antigen escape; the persistence and efficacy of CAR-T cells are enhanced by genetic modification or combined use of immune checkpoint inhibitors. Although CAR-T therapy has a significant effect, it may also cause side effects such as cytokine release syndrome (CRS) and immune effector cell-related neurotoxicity syndrome (ICANS).

### 3.4 Vaccine

The cancer vaccines currently studied mainly include Peptide vaccines and dendritic cell (DC) vaccines (133) (**Table 2**). Peptide vaccines mainly target leukemia-associated antigens including nephroblastoma 1 (WT1), protease 3 (PR3), hyaluronic acid-mediated motor receptor (RHAMM), and mucin 1 (MUC1) (134). Mutations in the WT1 gene may cause abnormalities in cell growth and differentiation, which play an important role in the occurrence of leukemia (135). At the same time, it has been reported that the overexpression of WT1-specific T cells is significantly associated with a variety of hematological diseases (136). In AML patients, humoral immune response and cytotoxic response to WT1 protein have been observed (137). The above findings provide a theoretical basis for the application of WT1 protein in cancer vaccine therapy. The multivalent WT1 peptide vaccine galinpepimut-S (GPS) is considered to induce a specific immune response and is associated with the 5-year survival rate of AML patients participating in this clinical trial (138). CV-501 is a different HLA class II restricted peptide vaccine based on WT1, which has been studied in patients with acute myeloid leukemia (AML). However, the test results of this vaccine did not show any significant immune response (139). High-dose RHAMM-R3 peptide vaccine can effectively stimulate the body ‘s immune response and has a good effect in the treatment of hematological malignancies (140). Studies have explored the effects of combined use of PADRE, adjuvants, and WT1 or PR3 vaccines in AML patients. The results showed that the combination failed to produce clinical or immune responses (141).

**Table 2:** Application of cancer vaccine in AML clinical trials.

Study identifier	Phase	Start dates	Status	Target	Study population	Number (n)	Study Type	clinical outcome
NCT00433745	II	2007-02	Completed	WT1	MDS,AML,ALL,CML	4	Interventional	All 4 patients had rapid relapses despite vaccination
NCT01266083	II	2011-01-14	Completed	WT1	AML,ALL	22	Interventional	Overall survival: Of the 22 patients, 19 (86.4%) were evaluable for survival at 3 years
NCT01773395	II	2013-01-08	Terminated		MDS,CMML,AML	123	Interventional	The results of the current study showed no improvement in Progression Free Survival or Overall Survival at 18 months after HSCT with GVAX vs placebo.
NCT00488592	II	2007-06	Completed	WT1,PR1	MDS,CML,AML	10	Interventional	Anti-leukemia responses can only occur when there are high-avidity leukemia-specific CD8+ T cells present. Additionally, repeated peptide vaccinations can result in the selective elimination of these high-avidity CD8+ T cells, leading to a decrease in anti-leukemia responses.
NCT00923910	I/II	2008-02-22	Completed	WT1	ALL,CML,AML,NHL	10	Interventional	
NCT02396134	II	2015-05-21	Active, not recruiting		MDS,CML,AML,	61	Interventional	
NCT02506933	II	2015-11-05	Active, not recruiting		MDS,CML,AML,HL,NHL	102	Interventional	Compared with the control group, the level of long-acting pp65-specific T cells with effective memory phenotype was significantly increased in Triplex.
NCT01611298	Not Applicable	2008-03	Completed		MDS,CML,ALL,AML,HL,NHL	7	Interventional	
NCT00398138	I	2006-10	Completed	WT-1	AML,MDS,NSCLC,Mesothelioma	22	Interventional	
NCT03761914	I/II	2019-09-30	Completed	WT-1	TNBC,AML,SCLC,mCRC,mOvC	26	Interventional	
NCT05000801	Not Applicable	2021-07-01	Recruiting	DC	AML	20	Interventional	
NCT01898663	I/II	2013-06	Recruiting	DC	AML	30	Interventional	
NCT01686334	II	2012-10	Active, not recruiting	DC	AML	130	Interventional	

Dendritic cells may be derived from different progenitor cells, but in the study of vaccine therapy, the most widely discussed sources are acute myeloid leukemia dendritic cells (AML-DC) (142) and monocyte-derived dendritic cells (mo-DC) (143). Most DC-based vaccine therapies are carried out in the state of minimal residual disease (MRD) to prevent the recurrence of leukemia. Leukemia-derived dendritic cell-based vaccines have shown good tolerance and a low probability of adverse reactions (144). Mo-DC can effectively load complete apoptotic leukemia cells, leukemia cell lysates or RNA/mRNA derived from leukemia cells, thereby further enhancing its therapeutic effect (145, 146). WT1 mRNA-electroporated DCs can improve the overall survival rate of AML patients with high risk of recurrence and further promote vaccine-induced WT1-specific CD8 T cell response (147). The efficacy of allogeneic dendritic cell vaccine DCP-001 in AML patients was studied. The results showed that DCP-001 had good safety and feasibility, and could induce cellular and humoral immune responses (148).

Telomerase is considered to be an important target in the study of cancer vaccine therapy. This is because the expression of telomerase is more in AML patients, and it plays a key role in maintaining the microenvironment of leukemia stem cells, especially in the context of high-risk cytogenetics. Studies have shown that telomerase (human telomerase reverse transcriptase, hTERT) and lysosomal-associated membrane protein (LAMP) can significantly enhance immunoreactivity by encoding mRNA, and this signal specifically targets lysosomes (149).

Exosome-based vaccines are a new approach to cancer treatment. The first study on tumor suppression using exosome-based vaccines was reported in 1998 (150). Some studies have evaluated the therapeutic effect of a novel interferon-modified exosome vaccine on prostate cancer (151). The inoculation of exosome vaccine not only reduces the expression level of vascular endothelial growth factor receptor 2, but also effectively reduces the ability of tumor metastasis (152). A study has shown that trastuzumab emtansine (T-DM1) can specifically bind to exosomes derived from HER2-positive cells, while some studies have explored the potential of exosomes as HER2-positive tumor vaccines by targeting and activating CD4 + and CD8 + T cells, and promoting long-term immune response through CTL memory cells (153, 154). Studies have found that exosomes derived from human adipose-derived mesenchymal stem cells (haMSC) can effectively induce apoptosis of ovarian cancer cells by blocking the cell cycle, up-regulating the molecular levels of BAX, CASP9 and CASP3, and down-regulating the anti-apoptotic protein BCL2 (155). A number of clinical studies have confirmed that vaccine therapy has shown significant efficacy in improving patient survival and reducing the risk of recurrence (156, 157). Leukemia-derived exosomes can induce the polarization of regulatory T cells and macrophages, and these exosomes promote the formation of tumor microenvironment in bone marrow, and activate HF-1α, AKT, VEGF, c-Myc, IL-8 and cyclin D1 signaling pathways by transporting tyrosine kinase receptor MET. AML-derived exosomes can transform the bone marrow microenvironment into conditions conducive to the development of leukemia by regulating multiple molecules (158). In addition, circulating exosomes produced by AML can also carry immunosuppressive substances, thereby inhibiting the body ‘s anti-tumor immune response (159). Exosomes can efficiently deliver drugs and antigens and accurately target malignant tumor cells in the blood system. At present, there are few reports on the application of MSC-derived exosome vaccines in acute myeloid leukemia.

## 4 Application of nanoimmunotherapy

Nano immunotherapy is an innovative targeted therapy in the field of cancer treatment. Nanotechnology refers to the use of biomolecules in the range of 5 to 500 nm in size for medical treatment and diagnosis. By improving the solubility and stability of drugs, prolonging the half-life of drugs in the blood, reducing toxic and side effects, and achieving precise targeting of drugs at specific sites, nanotechnology breaks the limitations of traditional therapies and gives full play to their anti-cancer effects (160–162). At present, nanomedicines for a variety of indications are being studied in clinical trials (163, 164). Nanoparticles (NP) used in nanopharmaceutical formulations currently cover a wide variety of types, including liposomes, polymers, micelles, nanocrystals, metal/metal oxides, and other inorganic materials and proteins (165). These NP can alter the biochemical, electronic, magnetic or optical properties of pharmaceutical preparations, allowing them to play an important role in therapeutic applications (166).

Compared with conventional immunotherapy alone, nanomedicines can enhance the immune response. By precisely targeting specific immune cells, the nanoparticle system activates and enhances their ability to recognize and attack cancer cells, thereby enhancing the immune system ‘s response (167). Targeting drug delivery is achieved by loading immune checkpoint inhibitors or immune stimulators onto NP, thereby enhancing the activation of immune cells and anti-tumor effects in TME. By loading tumor-specific antigens or immune stimulators onto NP to stimulate specific immune responses against tumors, thereby inhibiting tumor growth and metastasis, an effective cancer vaccine is prepared (168). Tumors usually lack a functional lymphatic system, which leads to long-term retention of macromolecules in tumors. This enhanced permeability and retention (EPR) effect is the main theoretical basis for current nano-drug design (169). With the help of the EPR effect, nanodrugs with a diameter greater than 8 nm can penetrate the blood vessel wall and enter the tumor cells, thereby achieving efficient drug delivery (170). Nano-diamonds can not only specifically target tumor cells, but also directly transport doxorubicin to mitochondria in cells, effectively cutting off the energy supply of cells, thereby inhibiting the growth and reproduction of tumor cells without affecting the function of normal cells (171).

The effect of cancer treatment can be significantly enhanced by using NP-based treatments. Nanogels enhance the anti-tumor effect by promoting the secretion of dendritic cells, cytotoxic T lymphocytes and immune stimulating cytokines, thereby improving the therapeutic effect on melanoma (172). In combination with anti-PD-L1 antibody therapy, the self-assembled core-shell nanosystem loaded with oxaliplatin and dihydroartemisinin can effectively induce T cell activation and reduce inhibitory cell infiltration in a mouse colorectal tumor model, thereby achieving a lasting enhancement of anti-tumor immunity (173). Studies have found that when α-PD-L1 and nanoparticles were injected into mice with lung cancer, the expression of IL-12 was significantly increased, while the levels of IL-10, arginase I and CCL22 were decreased, and the number of TREG cells was reduced, thus effectively inhibiting tumor growth (174). It has been reported that the use of PD-L1 siRNA-loaded folic acid (FA) modified polyethyleneimine nanoparticles can enhance the uptake of nanoparticles by ovarian cancer cells (175). As a carrier of mRNA vaccine, lipid nanoparticles (LNP) can not only efficiently deliver mRNA vaccine in liver tumor mouse model, but also stimulate specific immune response against tumor antigens (176, 177). Studies have shown that half of patients with unresectable pancreatic ductal adenocarcinoma (PDAC) showed a significant ability to induce antigen-specific T cells by using a novel personalized antigen vaccine developed by uridine-modified mRNA-lipid nanoparticle technology (178). The combination of CAR-T cell therapy and nanoparticles can improve the anti-tumor effect and enhance the targeting ability in cancer treatment, which provides a new research direction for the treatment of hepatocellular carcinoma (179). It has been reported that a NLS peptide-functionalized gold nanoparticle loaded with AS1411 and anti-221 can accurately bind AS1411 and anti-221 *in vitro* and *in vivo*, and target the key molecules in the NCL/miR-221/NFκB/DNMT1 signaling pathway, thereby effectively inhibiting the growth of AML cells (180). CPX-351 is a classic nanoliposome. Compared with traditional therapy, its toxicity to normal cells is significantly reduced, and it has a lower IC50 value. In addition, CPX-351 can promote the accumulation of daunorubicin and cytarabine in patients with acute myeloid leukemia (AML), thereby effectively improving the therapeutic effect of anti-leukemia (181, 182). The drug was approved by the US Food and Drug Administration (FDA) in 2017 for the treatment of newly diagnosed treatment-related AML (t-AML) or AML patients with myelodysplastic changes (183). The researchers developed a nanoparticle based on poly (lactic-co-glycolic acid) (PLGA), which was loaded with idarubicin (IDA) and achieved sustained release of IDA by methoxypolyethylene glycol-b-PLGA (mPEG-PLGA) technology. This design not only maintains the stable release of IDA, but also increases its anti-leukemia activity by 2 to 4 times compared to free IDA (184). In addition, studies have pointed out that by combining PLGA nanoparticles with anti-d44 antibody to form PLGA-antid44-PTL complexes and encapsulating parthenolide, an effective nuclear factor kappa B (NF-κB) inhibitor, the cellular uptake efficiency of drugs on acute myeloid leukemia cells can be significantly improved, thereby more effectively inhibiting the proliferation of these cells (185). Researchers have successfully developed an innovative ferritin dendrimer nanoparticle for precise delivery of miRNA to NB4 cells overexpressing the CD71 receptor. This technology can not only significantly induce leukemia cells to show phenotypic and morphological changes similar to early differentiation, but also effectively inhibit the cytotoxicity caused by free PAMAM dendrimers, and ensure the stability of nucleic acid during transmission to avoid its degradation (186). It is worth noting that miR-150, as a key tumor suppressor, plays an important role by negatively regulating FLT3. Experiments have shown that when PAMAM dendrimers are combined with FLT3 ligands and loaded with miR-150, selective clearance of FLT3-overexpressing acute myeloid leukemia (AML) cells can be achieved, and extremely low side effects are shown *in vivo* (187).

## 5 Conclusion

In this review, we summarize the progress of conventional immunotherapy and nanoimmunotherapy for AML, and highlight several representative emerging strategies (**Table 3**). AML is regarded as a disease with poor prognosis. In recent years, significant progress has been made in the molecular mechanism of tumor immunology and the clinical application of immunomodulators, which brings new hope for the treatment of AML. Because AML cells have immune escape characteristics, it is difficult for the immune system to effectively identify and attack these cancer cells. With the deepening understanding of tumor immunology, it provides an important theoretical basis for the development of more effective AML treatment. In order to improve the efficacy of immunotherapy for AML, we need to identify drug resistance mechanisms as early as possible and formulate corresponding strategies to overcome these mechanisms, thereby reducing the recurrence rate. Although there are few reports on the application of nano immunotherapy in AML, this field shows great potential and is expected to become a research hotspot in the near future. The current research mainly focuses on the combination therapy strategy, which shows a good development prospect in the future.

**Table 3:** FDA approved drugs for the treatment of hematological malignancies.

Drugs	Cancer type
Midostaurin Novartis	Treatment of adult patients with newly diagnosed AML who are FLT3+-in combination with standard cytarabine and daunorubicin induction and cytarabine consolidation
Enasidenib Celgene	Treatment of adult patients with relapsed or refractory AML with an isocitrate dehydrogenase-2 (IDH2) mutation
CPX-351 Jazz Pharmaceuticals	Treatment of adults with newly diagnosed therapy-related AML (t-AML) or AML with myelodysplasia related changes (AML-MRC)
Gemtuzumab ozogamicin Pfizer	Treatment of adults with newly diagnosed CD33-ositive AML and for treatment of relapsed or refractory CD33-positive AML in adults and in pediatric patients 2 years and older. May be used in combination with daunorubicin and cytarabine for adults with newly diagnosed AML.
Ivosidenib Agios	Adult patients with relapsed or refractory AL with a susceptible IDH1 mutation-
Glasdegib Pfizer	In combination with low-dose cytarabine for the treatment of newly diagnosed AML in adults who are aged 75 years or older, or who have comorbidities that preclude use of intensive induction chemotherapy.
Venetoclax Abbvie/Genetech	In combination with azacitidine or decitabine or low-dose cytarabine for the treatment of newly diagnosed AML in adults who are aged 75 years or older, or who have comorbidities that preclude use of intensive induction chemotherapy.
Gilteritinib Astellas Pharma	Treatment of adult patients who have relapsed or refractory AML with a FLT3 mutation-
idecabtagene vicleucel	Treatment of adult patients with relapsed or refractory multiple myeloma after two or more prior lines of therapy including an immunomodulatory agent, a proteasome inhibitor, and an anti-CD38 monoclonal antibody.
